# FLInt in *C. briggsae* and *C. tropicalis* : Strains and resources for fast and locus-targeted integration of multi-copy transgenes.

**DOI:** 10.17912/micropub.biology.002310

**Published:** 2026-08-26

**Authors:** Itai Antoine Toker, Oliver Hobert

**Affiliations:** 1 Department of Biological Sciences, Columbia University, New York, New York, USA; 2 Howard Hughes Medical Institute

## Abstract

*C. briggsae* and *C. tropicalis* are satellite model systems for molecular mechanistic investigations and for comparative studies across *Caenorhabditis* species. FLInt ( F luorescent L andmark Int erference) was developed in *C. elegans* for rapid generation of transgenic strains through integration of multi-copy DNA arrays into predetermined genomic loci, without requiring a random mutagenesis step. Here, we present resources and newly generated strains that enable FLInt in *C. briggsae* and *C. tropicalis* , yielding integrated transgenic lines within ~10 days of microinjection.

**Figure 1. Landing sites, strains and workflow for FLInt transgenics in C. briggsae and C. tropicalis:** ( **A-B** ) Genomic loci and synteny of the novel *tdTomato* landing sites on chromosome II ( **A** ) and V ( **B** ) in *C. briggsae* and *C. tropicalis* . Orthologs and paralogs bear the same color across species. ( **C** ) Representative fluorescent microscopy image of a *C. tropicalis* L4 individual of the landing site strain OH20371 ( **D** ) *C. tropicalis* (L4) FLInt-generated strain expressing the integrated transgene otIs1014[unc-31p::mScarlet3::NLS ; unc-31p::gfp::NLS *otSi9] V (green channel is shown). Upon integration, the ubiquitous *eft-3p::tdTomato::h2b* signal is lost and the new transgene is transmitted to 100% of progeny. Scale bars, 100 μm.( **E** ) Workflow for FLInt transgenics and strain isolation.

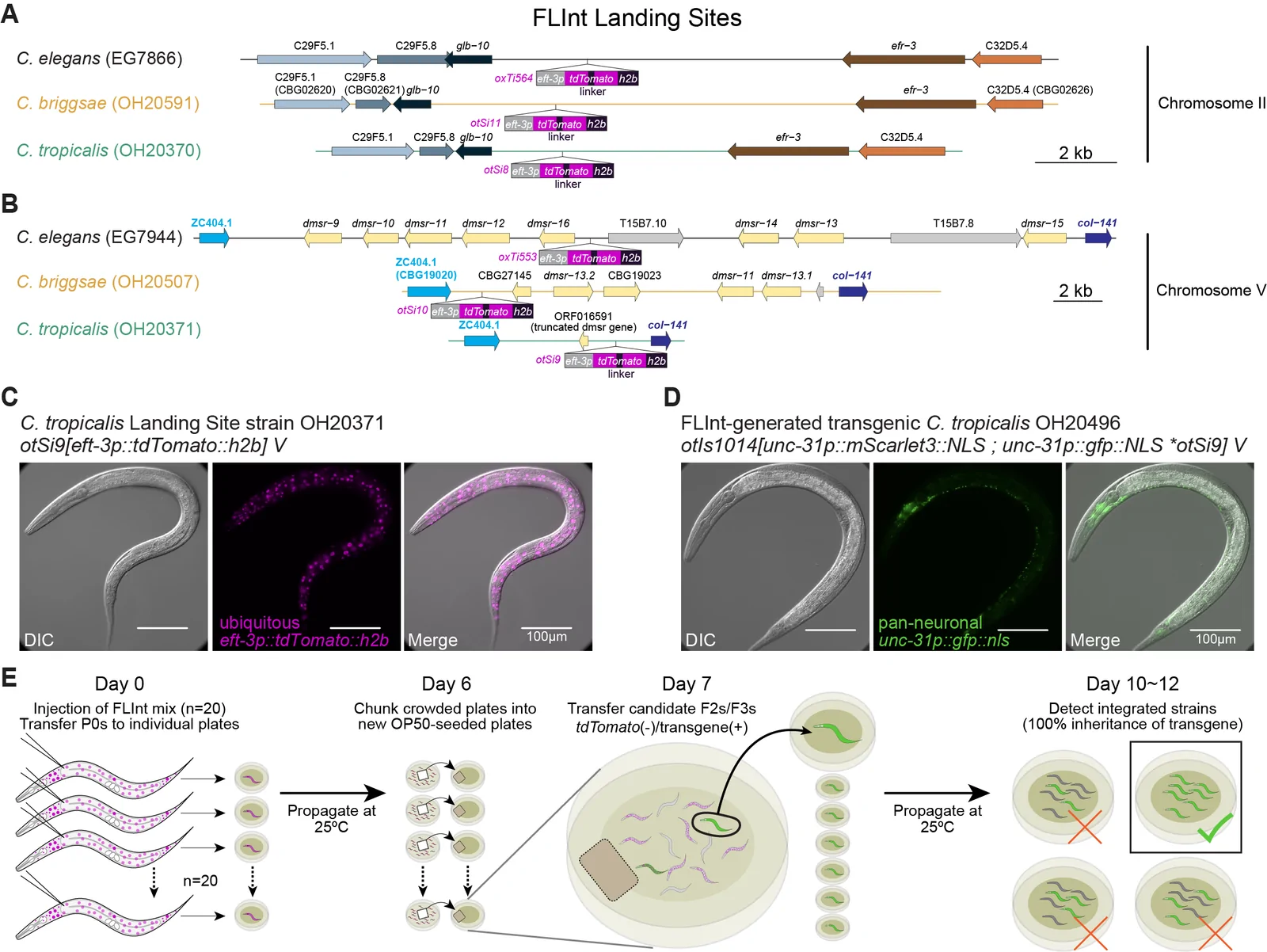



## Description

The nematodes *Caenorhabditis* *briggsae* and *Caenorhabditis* *tropicalis* are increasingly popular satellite model organisms. Their advantages for mechanistic studies have provided the foundation for research in diverse topics such as host-virus interactions, horizontal gene transfer, ecological adaptation and selfish genetic elements (Félix et al., 2011; Widen et al., 2023; Wang et al., 2026; Ross et al., 2011; Ben-David et al., 2021; Pliota et al., 2024). Moreover, their evolutionary position relative to *C. elegans* makes them prime systems for comparative studies, providing insight into the evolution of reproductive strategies, sex determination, cell lineages, responses to biotic and abiotic stress, neurodifferentiation and neuronal signaling (Wei et al., 2014; Shen et al., 2024; Zhao et al., 2008; Poullet et al., 2015; Richaud et al., 2026; Jhaveri et al., 2025; Toker et al., 2025).

CRISPR-based genome editing has been adapted to *C. briggsae* and *C. tropicalis* , and miniMos and bombardment techniques for generation of single- or low-copy insertions have been introduced (Zhao et al., 2010; Frøkjær-Jensen et al., 2014; Ding et al., 2022; Toker et al., 2025; Toker & Hobert, 2022). For fast generation of high-expressing transgenic strains, a popular approach has been the microinjection of DNA constructs which form extrachromosomal multi-copy arrays that can be transmitted to a subset of the progeny (Mello et al., 1991). However, many applications require multi-copy arrays to be integrated into the genome to prevent mosaicism and ensure full inheritance across generations.

Chromosomal integration of transgenes has historically been tedious and carried the substantial disadvantage of being highly mutagenic. Traditional methods (UV/TMP, gamma irradiation) rely on random genomic perturbations and preclude control over the insertion site (Nance & Frøkjær-Jensen, 2019). As a result, integrated strains must be backcrossed extensively to mitigate background mutations, while uncertainty about the integration locus complicates downstream applications. Recently, Malaiwong and colleagues developed FLInt ( ***F*** luorescent ***L*** andmark ***Int*** erference) in *C. elegans* to obtain fast integration of multi-copy transgenes into predetermined genomic loci with no random mutagenesis (Malaiwong et al., 2023, 2026). FLInt relies on pre-existing strains containing "Safe Harbor" landing sites expressing *tdTomato* (Frøkjær-Jensen et al., 2014) *.* The landing site serves multiple roles: it is the target for a CRISPR-Cas9-mediated DNA cut, the predetermined locus of transgene insertion, and an essential element of the screening strategy. Lines with successful integration lose *tdTomato* expression while stably inheriting expression of the inserted transgene of interest.

Here, we present a platform for FLInt transgenesis in *C. briggsae* and *C. tropicalis* . We used CRISPR-Cas9 to insert a single-copy *eft-3p::tdTomato::h2b::tbb-2 3'UTR* cassette into either chromosome II or chromosome V of strains AF16 ( *C. briggsae* ) and NIC203 ( *C. tropicalis* ), generating four independent strains with FLInt landing sites. We took advantage of the high synteny across *Caenorhabditis* species (Ben-David et al., 2021; Hillier et al., 2007) and chose genomic regions homologous to the *oxTi564* II and *oxTi553* V *C. elegans* loci (F **igure 1A-B** ). These two Safe Harbor landing sites are routinely used for efficient integration and expression of transgenes in *C. elegans* (Malaiwong et al., 2023; Sural et al., 2025). We confirmed that all four strains display ubiquitous nuclear expression of *tdTomato* that is easily detectable under a fluorescent dissecting microscope ( **Figure 1C** ). Of note, the single-copy *tdTomato* cassette used in the *C. briggsae* and *C. tropicalis* landing sites does not include the rescuing *Cbr-unc-119(+)* sequence present in the miniMos-generated *C. elegans* landing sites (Frøkjær-Jensen et al., 2014).

Starting with 20 injected P0 animals, we obtained at least one integrated line for all transgenes we attempted to date (3/3 in *C. briggsae* , 4/4 in *C. tropicalis* ) ( **Figure 1D** ). Our FLInt workflow (described in **Methods** ), which is based on published approaches with modifications (Malaiwong et al., 2023, 2026), permits the isolation of integrated strains ~10 days post-injection with minimal hands-on effort ( **Figure 1E** ). We generally use the original *tdTomato* FLInt crRNA guide 5'- GTGATGAACTTCGAGGACGG -3' (Malaiwong et al., 2023). However, due to sequence similarity between *tdTomato* and other commonly-used red fluorescent proteins (Shaner et al., 2004; Bindels et al., 2017), this crRNA may cross-react with some transgenes of interest. As an alternative, we used the crRNA 5'- CTCCTCCGAGGACAACAACA -3' that targets the linker region separating the *tdTomato* tandem repeats, and obtained integrated lines in both *C. briggsae* (1/1) and *C. tropicalis* (1/1). Both guides have been used successfully for FLInt in *C. elegans* , where their relative integration efficiencies have been examined in detail (Malaiwong et al., 2026).

More information on strain generation is provided in the Methods section. Strains generated in this study are made available through the *Caenorhabditis* Genetics Center (CGC).

## Methods

**Cultivation of nematodes**

*C. briggsae* and *C. tropicalis* animals were maintained at 25ºC on NGM plates seeded with OP50 *E. coli.*

**Selection of loci for novel *tdTomato* landing sites in *C. briggsae* and *C. tropicalis***

The strains EG7866 and EG7944 are reliable Safe Harbor strains for FLInt transgene integration in *C. elegans* (Malaiwong et al., 2023; Sural et al., 2025). For the presently described Safe Harbor strains in *C. briggsae* and *C. tropicalis* , we aimed to insert the *tdTomato* landing sites in genomic regions homologous to the landing sites in EG7866 ( *oxTi564* , chromosome II) and EG7944 ( *oxTi553* , chromosome V).

*oxTi564* (chromosome II) is located between the *C. elegans* genes *glb-10* and *efr-3* . The orthologs of *glb‑10* , *efr‑3* and their neighboring genes are found in similar configuration in all three species. The intergenic region between *glb-10* and *efr-3* was chosen for the landing sites in *C. briggsae* OH20591 and *C. tropicalis* OH20370. In **Figure 1A** , the genomic region in *C. briggsae* is shown in reverse orientation relative to the AF16 genome coordinates for ease of visualization.

*oxTi553* (chromosome V) is located in an intergenic zone in the vicinity of the *dmsr-* genes ( D ro M yo S uppressin Receptor related) *dmsr-9* to *dmsr-16* . This family of neuropeptide receptors is highly evolutionarily dynamic (Golinelli et al., 2024), and some *dmsr-* genes near *oxTi553* resulted from recent duplications. The area that includes *dmsr-9* -to *-16* and *oxTi553* is flanked by the genes ZC404.1 and *col-141* ( **Figure 1B** ). Similar to *C. elegans* , the ZC404.1 and *col-141* orthologs in *C. briggsae* and *C. tropicalis* are positioned on chromosome V and are separated by *dmsr-* or truncated *dmsr-* genes. Intergenic regions between the orthologs of ZC404.1 and *col-141* were chosen for landing site insertions in *C. briggsae* OH20507 and *C. tropicalis* OH20371.

genoPlotR (Guy et al., 2010) was used to visualize the genomic regions in Figure 1A- B.

**Genomic insertion of FLInt landing sites in *C. briggsae* and *C. tropicalis***

We inserted single-copy *eft-3p::tdTomato::h2b::tbb-2 3'UTR* landing sites through CRISPR-Cas9 as described in (Toker et al., 2025), using long ssODN repair templates prepared according to (Eroglu et al., 2023) and injection mix concentrations from (Ghanta & Mello, 2020).

To generate ssODN CRISPR repair templates, we used long primers to PCR-amplify (Q5 reagents, NEB M0491) the insertion sequence flanked by 35 bp homology arms from the plasmid pIAT034 containing the *eft-3p::tdTomato::h2b::tbb-2 3'UTR* sequence. For these PCRs, one primer (but not both) included a phosphorylated 5' nucleotide. PCRs were run in 200μl reactions (4 side-by-side replicates of 50 μl), confirmed using agarose gel electrophoresis, pooled and column-purified (Invitrogen™ PureLink™ #K310001). Eluted purified PCR products (45 μl in nuclease-free water) were digested into single-stranded DNA using lambda exonuclease (NEB M0262L) and accompanying buffer for 20 minutes at 37ºC (total reaction volume 50 μl). Digestion was followed by column purification using the Monarch® system (NEB T3010S) and elution with 6 μl nuclease-free water.

To prepare the injection mix, Cas9 (0.5 μl of 10 μg/μl stock, IDT #1081059), tracrRNA (5 μl of 0.4 μg/μl stock, IDT #1072532) and crRNA (2.8 μl of 0.4 μg/μl stock) were first mixed and incubated at 37ºC for 15 minutes. Then, ssODN (2.2 μg) was added, complemented with nuclease-free water to a final volume of 20 μl. Mixes were injected to 15 P0 young adult *C. briggsae* (strain AF16) or *C. tropicalis* (strain NIC203) nematodes, which were then maintained at 25ºC. 4 days post-injection, the F1 progeny were screened under a fluorescent dissecting microscope (Leica M165FC), and candidate heterozygotes selected based on the expected *eft-3p* :: *tdTomato::h2b* fluorescence pattern (ubiquitous nuclear). Homozygote strains were isolated in the following generations. Landing site insertions were confirmed via PCR and long-read sequencing.

**FLInt injection mix**

In FLInt injections, the mix includes the linearized transgene(s) of interest together with CRISPR-Cas9 reagents that target the *tdTomato* landing site for DNA cleavage.

1) tracrRNA (5 μl of 0.4 μg/μl stock, IDT #1072532) and *tdTomato* -targeting crRNA (2.8 μl of 0.4 μg/μl stock) are mixed together in an RNAse-free PCR tube.

2) Mixture is transferred to a PCR thermocycler for a denaturation-annealing step:

- 5 min at 95ºC.

- 5 min at 10ºC.

3) Annealed crRNA::tracrRNA mix is transferred into an RNAse-free Eppendorf tube containing Cas9 (0.5 μl of 10 μg/μl stock, IDT #1081059).

4) Mix is incubated for 15 min at 37ºC.

5) Ribonucleoprotein mixture is moved to ice. Then, transgenes of interest (PCR products, linearized plasmids and/or co-injection markers) are added at desired concentration supplemented with DNA ladder (GeneRuler 1kb Plus DNA Ladder, Thermo Scientific ^TM^ SM1332) and nuclease-free water, to reach a total DNA concentration of 100 ng/μl in a 20 μl mix volume.

For the transgenic line shown in ( **Figure 1D** ), plasmids pMZ1037 and pMZ1180 were linearized (restriction enzyme PvuI with rCutsmart buffer, NEB R3150L), column purified and injected at a concentration of 2.5 ng/μl each, with 95 ng/μl DNA ladder and the *tdTomato* CRISPR-Cas9 reagents.

**FLInt workflow and isolation of integrated transgenic strains**

Day 0 : Inject FLInt mix into 20 P0 young adults. Transfer P0s individually into separate OP50-seeded 60 mm NGM plates and maintain worms at 25ºC for 5~6 days to propagate progeny for 2+ generations.

Day 6-7 : Once the plates are crowded with worms and devoid of OP50, transfer a chunk from each plate into new OP50-seeded plates. Let the F2/F3 larvae exit the chunk and disperse in the bacterial lawn for at least 3h to aid in single worm transfer (16h~24h for most convenient screening). Under a fluorescent dissecting microscope, screen for candidate F2s/F3s expressing the transgenic arrays and lacking the ubiquitous nuclear *tdTomato* signal. Single the candidates by transferring them individually to new seeded plates. Prioritize chunks that display multiple (>5) candidate hits, and single multiple candidates from those plates, as they are more likely to have been derived from integrated individuals. Keep track of the P0 of origin of singled candidates.

Day 10-12 : Inspect plates and select strains with 100% transmission of the transgene (and absence of ubiquitous nuclear *tdTomato* signal). Successful strains that derived from distinct P0s are independent transgenic lines.

**Imaging**

L4 individuals were mounted on a glass slide padded with a 5% agarose patch and anesthetized in a drop of M9 with 50 mM sodium azide. Images were acquired using a Zeiss Axio Imager.Z2 compound microscope.

## Reagents

**Plasmids**

**Table:** 

**Name**	**Description**	**Source**
pIAT034	*eft-3p::tdTomato::h2b::tbb-2 3' UTR*	This study
pMZ1037	*unc-31p::mScarlet3::NLS::unc-54 3' UTR*	(Fieseler et al., 2025)
pMZ1180	*unc-31p::gfp::NLS::unc-54 3' UTR*	(Fieseler et al., 2025)

**Nematode strains**

**Table:** 

**Species**	**Strain**	**Genotype**	**Description**
*C. briggsae*	AF16		wild isolate
*C. tropicalis*	NIC203		wild isolate
*C. briggsae*	OH20591	*otSi11[eft-3p::tdTomato::h2b::tbb-2 3'UTR] II*	Landing site on chromosome II, *C. briggsae*
*C. briggsae*	OH20507	*otSi10[eft-3p::tdTomato::h2b::tbb-2 3'UTR]* V	Landing site on chromosome V, *C. briggsae*
*C. tropicalis*	OH20370	*otSi8[eft-3p::tdTomato::h2b::tbb-2 3'UTR]* II	Landing site on chromosome II, *C. tropicalis*
*C. tropicalis*	OH20371	*otSi9[eft-3p::tdTomato::h2b::tbb-2 3'UTR]* V	Landing site on chromosome V, *C. tropicalis*
*C. tropicalis*	OH20496	otIs1014[unc-31p::mScarlet3::NLS::unc-54 3'UTR; unc-31p::gfp::NLS::unc-54 3'UTR *otSi9] V	pan-neuronal nuclear *gfp* and *mScarlet3* transgene integrated into *otSi9* locus

***tdTomato* crRNAs for FLInt**

Original crRNA (cuts *tdTomato* twice): 5'-GTGATGAACTTCGAGGACGG-3'

Alternative crRNA ( *tdTomato* linker region): 5'-CTCCTCCGAGGACAACAACA -3'

**crRNAs for generation of landing sites**

- ***C. briggsae* chromosome II - *otSi11***

crRNA_IAT080 5'-AATTGGCAGATGGCACAGAA-3'

- ***C. briggsae* chromosome V - *otSi10***

crRNA_IAT081 5'-ATGTAGTAAAACGGGTGACA-3'

- ***C. tropicalis* chromosome II – *otSi8***

crRNA_IAT074 + crRNA_IAT075 (injected together)

5'-CAACTGGAAAAGCCTAATTG-3' + 5'-TTGAGGGAGTCAACACAGAA-3'

- ***C. tropicalis* chromosome V – *otSi9***

crRNA_IAT078 + crRNA_IAT079 (injected together)

5'-CTGATTCTCTGGATATTCGT-3' + 5'-TCTGATTCTCTGGATATTCG-3'

**Flanking regions of landing sites (40 nucleotides on each side)**

- ***C. briggsae* chromosome II - *otSi11***

5'-TGATGGTCATCCAGAGACATCTTCTTCTTCTTTTCCGTTC[ ***otSi11*** ]TGTGCCATCTGCCAATTAGGCTTCTCCGTCCCCAAAACTC-3'

- ***C. briggsae* chromosome V - *otSi10***

5'-TTAATGAGGAAGGAAAGTTAGGAATGTAGTAAAACGGGTG[ ***otSi10*** ]ACATGGTATCCGTCTAGTCTCTAGAATAGAGGAACACAGG-3'

- ***C. tropicalis* chromosome II – *otSi8***

5'-AATGTGTTTTGTGAGAAGACACGCAACTGGAAAAGCCTAA[ ***otSi8*** ]GAAC **c** GAAAATATAGAAAAAGGACAAGAAAGAGAAGAAGA-'3

lowercase “c” in fifth position downstream of *otSi8* was mutated to disrupt a PAM sequence.

- ***C. tropicalis* chromosome V – *otSi9***

5'-CTGGTCATTGGAATTGCAGAACCAGTTGTGATTCCCACGA[ ***otSi9*** ]ATATCCAGAGAATCAGAGCAATTATGAGAGTATACTTCCT-3'

**ssODNs for landing sites**

- ***C. briggsae* chromosome II - *otSi11*** *–* ***ssODN_IAT066***

5'-GTTAGGGGGAGGCACGCAACGAGTTTTGGGGACGGAGAAGCCTAATTGGCAGATGGCACAgatagcattcacttcactcagatgcaaacttttcagtgactataaaaagtcataaaataataacattaaaaaagcaaataaattagcgagagaattttttgacaaaaagaaagaagagtgatagagaagaagggaatgcttgaaaggatcttgcatTCACTTGCTGGAAGTGTACTTGGTAACGGCCTTGGTTCCCTCAGACACGGCGTGCTTGGCAAGCTCTCCTGGAAGGATCAGACGGACAGCGGTCTGAATTTCGCGGGATGAGATTGTGGAACGCTTGTTGTAGTGAGCAAGACGGGATGCTTCAGCAGCAATACGCTCGAAGACATCGTTGACAAAAGAGTTCATGATAGACATGGCTTTGGAGGAAACTCCAGTGTCTGGATGAACTTGCTTGAGGACACGGTAGATGTAGACGGAGTATGATTCCTTACGGGCATGACGTCTCTTCTTTCCGTCCTTTGGCTTCGTAACGGTCTTGGCGGCCTTCTTGGCTCCCTTGGCAGATGGCTTTGGTGGACTAGTTGCCCGGGCGGATCCCTCCACTTTGTACAAGAAAGCTGGGTACTTGTACAGCTCGTCCATGCCGTACAGGAACAGGTGGTGGCGGCCCTCGGAGCGCTCGTACTGTTCCACGATGGTGTAGTCCTCGTTGTGGGAGGTGATGTCCAGCTTGGTGTCCACGTAGTAGTAGCCGGGCAGTTGCACGGGCTTCTTGGCCATGTAGATGGTCTTGAACTCCACCAGGTAGTGGCCGCCGTCCTTCAGCTTCAGGGCCTGGTGGATCTCGCCCTTCAGCACGCCGTCGCGGGGGTACAGGCGCTCGGTGGAGGCCTCCCAGCCCATGGTCTTCTTCTGCATTACGGGGCCGTCGGGGGGGAAGTTGGTGCCGCGCATCTTCACCTTGTAGATCAGCGTGCCGTCCTGCAGGGAGGAGTCCTGGGTCACGGTCACCAGACCGCCGTCCTCGAAGTTCATCACGCGCTCCCACTTGAAGCCCTCGGGGAAGGACAGCTTCTTGTAATCGGGGATGTCGGCGGGGTGCTTCACGTACGCCTTGGAGCCGTACATGAACTGGGGGGACAGGATGTCCCAGGCGAAGGGCAGGGGGCCGCCCTTGGTCACCTTCAGCTTGGCGGTCTGGGTGCCCTCGTAGGGGCGGCCCTCGCCCTCGCCCTCGATCTCGAACTCGTGGCCGTTCATGGAGCCCTCCATGCGCACCTTGAAGCGCATGAACTCTTTGATGACGGCCATGTTGTTGTCCTCGGAGGAGGCGGTGCCGGAGCTGCCGCTGCCGGTGCTGCCGGTGCCATGCCCCAGGAACAGGTGGTGGCGGCCCTCGGAGCGCTCGTACTGTTCCACGATGGTGTAGTCCTCGTTGTGGGAGGTGATGTCCAGCTTGGTGTCCACGTAGTAGTAGCCGGGCAGTTGCACGGGCTTCTTGGCCATGTAGATGGTCTTGAACTCCACCAGGTAGTGGCCGCCGTCCTTCAGCTTCAGGGCCTGGTGGATCTCGCCCTTCAGCACGCCGTCGCGGGGGTACAGGCGCTCGGTGGAGGCCTCCCAGCCCATGGTCTTCTTCTGCATTACGGGGCCGTCGGGGGGGAAGTTGGTGCCGCGCATCTTCACCTTGTAGATCAGCGTGCCGTCCTGCAGGGAGGAGTCCTGGGTCACGGTCACCAGACCGCCGTCCTCGAAGTTCATCACGCGCTCCCACTTGAAGCCCTCGGGGAAGGACAGCTTCTTGTAATCGGGGATGTCGGCGGGGTGCTTCACGTACGCCTTGGAGCCGTACATGAACTGGGGGGACAGGATGTCCCAGGCGAAGGGCAGGGGGCCGCCCTTGGTCACCTTCAGCTTGGCGGTCTGGGTGCCCTCGTAGGGGCGGCCCTCGCCCTCGCCCTCGATCTCGAACTCGTGGCCGTTCATGGAGCCCTCCATGCGCACCTTGAAGCGCATGAACTCTTTGATGACCTCCTCGCCCTTGCTCACCATttttgagcctgcttttttgtacaaacttgtgagcaaagtgtttcccaactgaaaaaaaaacaatttaatttaaaagtaagaagagtgcggacggtagagagaataaaagtcgaaaaaattatcctagaaacttctaatatttttaaaaattaaaaaaatataaaaagtgaggtcacaaataagacagttagaacatttgttgaaagcctaaaataaaaattcgactaaacagtgagaacaggaaaaacgctgggactgaatgtttgacctcattataaattcataaaatttaaatttcctaatctggataaaaacaaagataaaaacaaaataaaccaacaaaaaaccaacatgattagtcagatgaccagaaaactcacgttaatttcacaaggcccaaaaaaatctctccccctctcgttgctgcctgcacatctaactcctagcacgaaaatgtaccgtacaccatttcaaacactctcggcgggagtgttgcatactttttctctctggcagtttattttcaataaattcttcatttttatattgtataatgtctttattttatgaaaaattcattaatttaacagaaacaatggaagaaccaatggaagttgacaataaaagaccaaaggtgcGAACGGAAAAGAAGAAGAAGATGTCTCTGGATGACCATCAGTTGTTTCGGTTTTGGTTCT-3'

- ***C. briggsae* chromosome V - *otSi10*** *–* ***ssODN_IAT067***

5'-ACTACTGAAAGTGGGAGGAATTAATGAGGAAGGAAAGTTAGGAATGTAGTAAAACGGGTGgcacctttggtcttttattgtcaacttccattggttcttccattgtttctgttaaattaatgaatttttcataaaataaagacattatacaatataaaaatgaagaatttattgaaaataaactgccagagagaaaaagtatgcaacactcccgccgagagtgtttgaaatggtgtacggtacattttcgtgctaggagttagatgtgcaggcagcaacgagagggggagagatttttttgggccttgtgaaattaacgtgagttttctggtcatctgactaatcatgttggttttttgttggtttattttgtttttatctttgtttttatccagattaggaaatttaaattttatgaatttataatgaggtcaaacattcagtcccagcgtttttcctgttctcactgtttagtcgaatttttattttaggctttcaacaaatgttctaactgtcttatttgtgacctcactttttatatttttttaatttttaaaaatattagaagtttctaggataattttttcgacttttattctctctaccgtccgcactcttcttacttttaaattaaattgtttttttttcagttgggaaacactttgctcacaagtttgtacaaaaaagcaggctcaaaaATGGTGAGCAAGGGCGAGGAGGTCATCAAAGAGTTCATGCGCTTCAAGGTGCGCATGGAGGGCTCCATGAACGGCCACGAGTTCGAGATCGAGGGCGAGGGCGAGGGCCGCCCCTACGAGGGCACCCAGACCGCCAAGCTGAAGGTGACCAAGGGCGGCCCCCTGCCCTTCGCCTGGGACATCCTGTCCCCCCAGTTCATGTACGGCTCCAAGGCGTACGTGAAGCACCCCGCCGACATCCCCGATTACAAGAAGCTGTCCTTCCCCGAGGGCTTCAAGTGGGAGCGCGTGATGAACTTCGAGGACGGCGGTCTGGTGACCGTGACCCAGGACTCCTCCCTGCAGGACGGCACGCTGATCTACAAGGTGAAGATGCGCGGCACCAACTTCCCCCCCGACGGCCCCGTAATGCAGAAGAAGACCATGGGCTGGGAGGCCTCCACCGAGCGCCTGTACCCCCGCGACGGCGTGCTGAAGGGCGAGATCCACCAGGCCCTGAAGCTGAAGGACGGCGGCCACTACCTGGTGGAGTTCAAGACCATCTACATGGCCAAGAAGCCCGTGCAACTGCCCGGCTACTACTACGTGGACACCAAGCTGGACATCACCTCCCACAACGAGGACTACACCATCGTGGAACAGTACGAGCGCTCCGAGGGCCGCCACCACCTGTTCCTGGGGCATGGCACCGGCAGCACCGGCAGCGGCAGCTCCGGCACCGCCTCCTCCGAGGACAACAACATGGCCGTCATCAAAGAGTTCATGCGCTTCAAGGTGCGCATGGAGGGCTCCATGAACGGCCACGAGTTCGAGATCGAGGGCGAGGGCGAGGGCCGCCCCTACGAGGGCACCCAGACCGCCAAGCTGAAGGTGACCAAGGGCGGCCCCCTGCCCTTCGCCTGGGACATCCTGTCCCCCCAGTTCATGTACGGCTCCAAGGCGTACGTGAAGCACCCCGCCGACATCCCCGATTACAAGAAGCTGTCCTTCCCCGAGGGCTTCAAGTGGGAGCGCGTGATGAACTTCGAGGACGGCGGTCTGGTGACCGTGACCCAGGACTCCTCCCTGCAGGACGGCACGCTGATCTACAAGGTGAAGATGCGCGGCACCAACTTCCCCCCCGACGGCCCCGTAATGCAGAAGAAGACCATGGGCTGGGAGGCCTCCACCGAGCGCCTGTACCCCCGCGACGGCGTGCTGAAGGGCGAGATCCACCAGGCCCTGAAGCTGAAGGACGGCGGCCACTACCTGGTGGAGTTCAAGACCATCTACATGGCCAAGAAGCCCGTGCAACTGCCCGGCTACTACTACGTGGACACCAAGCTGGACATCACCTCCCACAACGAGGACTACACCATCGTGGAACAGTACGAGCGCTCCGAGGGCCGCCACCACCTGTTCCTGTACGGCATGGACGAGCTGTACAAGTACCCAGCTTTCTTGTACAAAGTGGAGGGATCCGCCCGGGCAACTAGTCCACCAAAGCCATCTGCCAAGGGAGCCAAGAAGGCCGCCAAGACCGTTACGAAGCCAAAGGACGGAAAGAAGAGACGTCATGCCCGTAAGGAATCATACTCCGTCTACATCTACCGTGTCCTCAAGCAAGTTCATCCAGACACTGGAGTTTCCTCCAAAGCCATGTCTATCATGAACTCTTTTGTCAACGATGTCTTCGAGCGTATTGCTGCTGAAGCATCCCGTCTTGCTCACTACAACAAGCGTTCCACAATCTCATCCCGCGAAATTCAGACCGCTGTCCGTCTGATCCTTCCAGGAGAGCTTGCCAAGCACGCCGTGTCTGAGGGAACCAAGGCCGTTACCAAGTACACTTCCAGCAAGTGAatgcaagatcctttcaagcattcccttcttctctatcactcttctttctttttgtcaaaaaattctctcgctaatttatttgcttttttaatgttattattttatgactttttatagtcactgaaaagtttgcatctgagtgaagtgaatgctatcACATGGTATCCGTCTAGTCTCTAGAATAGAGGAACACAGGATATTGATTGGGTTGTACTT-3'

- ***C. tropicalis* chromosome II – *otSi8 – ssODN_IAT063***

5'-GAAATGATAAAGTACAAGAAAATGTGTTTTGTGAGAAGACACGCAACTGGAAAAGCCTAAgcacctttggtcttttattgtcaacttccattggttcttccattgtttctgttaaattaatgaatttttcataaaataaagacattatacaatataaaaatgaagaatttattgaaaataaactgccagagagaaaaagtatgcaacactcccgccgagagtgtttgaaatggtgtacggtacattttcgtgctaggagttagatgtgcaggcagcaacgagagggggagagatttttttgggccttgtgaaattaacgtgagttttctggtcatctgactaatcatgttggttttttgttggtttattttgtttttatctttgtttttatccagattaggaaatttaaattttatgaatttataatgaggtcaaacattcagtcccagcgtttttcctgttctcactgtttagtcgaatttttattttaggctttcaacaaatgttctaactgtcttatttgtgacctcactttttatatttttttaatttttaaaaatattagaagtttctaggataattttttcgacttttattctctctaccgtccgcactcttcttacttttaaattaaattgtttttttttcagttgggaaacactttgctcacaagtttgtacaaaaaagcaggctcaaaaATGGTGAGCAAGGGCGAGGAGGTCATCAAAGAGTTCATGCGCTTCAAGGTGCGCATGGAGGGCTCCATGAACGGCCACGAGTTCGAGATCGAGGGCGAGGGCGAGGGCCGCCCCTACGAGGGCACCCAGACCGCCAAGCTGAAGGTGACCAAGGGCGGCCCCCTGCCCTTCGCCTGGGACATCCTGTCCCCCCAGTTCATGTACGGCTCCAAGGCGTACGTGAAGCACCCCGCCGACATCCCCGATTACAAGAAGCTGTCCTTCCCCGAGGGCTTCAAGTGGGAGCGCGTGATGAACTTCGAGGACGGCGGTCTGGTGACCGTGACCCAGGACTCCTCCCTGCAGGACGGCACGCTGATCTACAAGGTGAAGATGCGCGGCACCAACTTCCCCCCCGACGGCCCCGTAATGCAGAAGAAGACCATGGGCTGGGAGGCCTCCACCGAGCGCCTGTACCCCCGCGACGGCGTGCTGAAGGGCGAGATCCACCAGGCCCTGAAGCTGAAGGACGGCGGCCACTACCTGGTGGAGTTCAAGACCATCTACATGGCCAAGAAGCCCGTGCAACTGCCCGGCTACTACTACGTGGACACCAAGCTGGACATCACCTCCCACAACGAGGACTACACCATCGTGGAACAGTACGAGCGCTCCGAGGGCCGCCACCACCTGTTCCTGGGGCATGGCACCGGCAGCACCGGCAGCGGCAGCTCCGGCACCGCCTCCTCCGAGGACAACAACATGGCCGTCATCAAAGAGTTCATGCGCTTCAAGGTGCGCATGGAGGGCTCCATGAACGGCCACGAGTTCGAGATCGAGGGCGAGGGCGAGGGCCGCCCCTACGAGGGCACCCAGACCGCCAAGCTGAAGGTGACCAAGGGCGGCCCCCTGCCCTTCGCCTGGGACATCCTGTCCCCCCAGTTCATGTACGGCTCCAAGGCGTACGTGAAGCACCCCGCCGACATCCCCGATTACAAGAAGCTGTCCTTCCCCGAGGGCTTCAAGTGGGAGCGCGTGATGAACTTCGAGGACGGCGGTCTGGTGACCGTGACCCAGGACTCCTCCCTGCAGGACGGCACGCTGATCTACAAGGTGAAGATGCGCGGCACCAACTTCCCCCCCGACGGCCCCGTAATGCAGAAGAAGACCATGGGCTGGGAGGCCTCCACCGAGCGCCTGTACCCCCGCGACGGCGTGCTGAAGGGCGAGATCCACCAGGCCCTGAAGCTGAAGGACGGCGGCCACTACCTGGTGGAGTTCAAGACCATCTACATGGCCAAGAAGCCCGTGCAACTGCCCGGCTACTACTACGTGGACACCAAGCTGGACATCACCTCCCACAACGAGGACTACACCATCGTGGAACAGTACGAGCGCTCCGAGGGCCGCCACCACCTGTTCCTGTACGGCATGGACGAGCTGTACAAGTACCCAGCTTTCTTGTACAAAGTGGAGGGATCCGCCCGGGCAACTAGTCCACCAAAGCCATCTGCCAAGGGAGCCAAGAAGGCCGCCAAGACCGTTACGAAGCCAAAGGACGGAAAGAAGAGACGTCATGCCCGTAAGGAATCATACTCCGTCTACATCTACCGTGTCCTCAAGCAAGTTCATCCAGACACTGGAGTTTCCTCCAAAGCCATGTCTATCATGAACTCTTTTGTCAACGATGTCTTCGAGCGTATTGCTGCTGAAGCATCCCGTCTTGCTCACTACAACAAGCGTTCCACAATCTCATCCCGCGAAATTCAGACCGCTGTCCGTCTGATCCTTCCAGGAGAGCTTGCCAAGCACGCCGTGTCTGAGGGAACCAAGGCCGTTACCAAGTACACTTCCAGCAAGTGAatgcaagatcctttcaagcattcccttcttctctatcactcttctttctttttgtcaaaaaattctctcgctaatttatttgcttttttaatgttattattttatgactttttatagtcactgaaaagtttgcatctgagtgaagtgaatgctatcGAACCGAAAATATAGAAAAAGGACAAGAAAGAGAAGAAGAAGAAGATGTCACTGGCTCATCAGTA-3'

- ***C. tropicalis* chromosome V – *otSi9 – ssODN_IAT065***

5'-TCAGTGAGAATTGGAAAAGCAGGAAGTATACTCTCATAATTGCTCTGATTCTCTGGATATgatagcattcacttcactcagatgcaaacttttcagtgactataaaaagtcataaaataataacattaaaaaagcaaataaattagcgagagaattttttgacaaaaagaaagaagagtgatagagaagaagggaatgcttgaaaggatcttgcatTCACTTGCTGGAAGTGTACTTGGTAACGGCCTTGGTTCCCTCAGACACGGCGTGCTTGGCAAGCTCTCCTGGAAGGATCAGACGGACAGCGGTCTGAATTTCGCGGGATGAGATTGTGGAACGCTTGTTGTAGTGAGCAAGACGGGATGCTTCAGCAGCAATACGCTCGAAGACATCGTTGACAAAAGAGTTCATGATAGACATGGCTTTGGAGGAAACTCCAGTGTCTGGATGAACTTGCTTGAGGACACGGTAGATGTAGACGGAGTATGATTCCTTACGGGCATGACGTCTCTTCTTTCCGTCCTTTGGCTTCGTAACGGTCTTGGCGGCCTTCTTGGCTCCCTTGGCAGATGGCTTTGGTGGACTAGTTGCCCGGGCGGATCCCTCCACTTTGTACAAGAAAGCTGGGTACTTGTACAGCTCGTCCATGCCGTACAGGAACAGGTGGTGGCGGCCCTCGGAGCGCTCGTACTGTTCCACGATGGTGTAGTCCTCGTTGTGGGAGGTGATGTCCAGCTTGGTGTCCACGTAGTAGTAGCCGGGCAGTTGCACGGGCTTCTTGGCCATGTAGATGGTCTTGAACTCCACCAGGTAGTGGCCGCCGTCCTTCAGCTTCAGGGCCTGGTGGATCTCGCCCTTCAGCACGCCGTCGCGGGGGTACAGGCGCTCGGTGGAGGCCTCCCAGCCCATGGTCTTCTTCTGCATTACGGGGCCGTCGGGGGGGAAGTTGGTGCCGCGCATCTTCACCTTGTAGATCAGCGTGCCGTCCTGCAGGGAGGAGTCCTGGGTCACGGTCACCAGACCGCCGTCCTCGAAGTTCATCACGCGCTCCCACTTGAAGCCCTCGGGGAAGGACAGCTTCTTGTAATCGGGGATGTCGGCGGGGTGCTTCACGTACGCCTTGGAGCCGTACATGAACTGGGGGGACAGGATGTCCCAGGCGAAGGGCAGGGGGCCGCCCTTGGTCACCTTCAGCTTGGCGGTCTGGGTGCCCTCGTAGGGGCGGCCCTCGCCCTCGCCCTCGATCTCGAACTCGTGGCCGTTCATGGAGCCCTCCATGCGCACCTTGAAGCGCATGAACTCTTTGATGACGGCCATGTTGTTGTCCTCGGAGGAGGCGGTGCCGGAGCTGCCGCTGCCGGTGCTGCCGGTGCCATGCCCCAGGAACAGGTGGTGGCGGCCCTCGGAGCGCTCGTACTGTTCCACGATGGTGTAGTCCTCGTTGTGGGAGGTGATGTCCAGCTTGGTGTCCACGTAGTAGTAGCCGGGCAGTTGCACGGGCTTCTTGGCCATGTAGATGGTCTTGAACTCCACCAGGTAGTGGCCGCCGTCCTTCAGCTTCAGGGCCTGGTGGATCTCGCCCTTCAGCACGCCGTCGCGGGGGTACAGGCGCTCGGTGGAGGCCTCCCAGCCCATGGTCTTCTTCTGCATTACGGGGCCGTCGGGGGGGAAGTTGGTGCCGCGCATCTTCACCTTGTAGATCAGCGTGCCGTCCTGCAGGGAGGAGTCCTGGGTCACGGTCACCAGACCGCCGTCCTCGAAGTTCATCACGCGCTCCCACTTGAAGCCCTCGGGGAAGGACAGCTTCTTGTAATCGGGGATGTCGGCGGGGTGCTTCACGTACGCCTTGGAGCCGTACATGAACTGGGGGGACAGGATGTCCCAGGCGAAGGGCAGGGGGCCGCCCTTGGTCACCTTCAGCTTGGCGGTCTGGGTGCCCTCGTAGGGGCGGCCCTCGCCCTCGCCCTCGATCTCGAACTCGTGGCCGTTCATGGAGCCCTCCATGCGCACCTTGAAGCGCATGAACTCTTTGATGACCTCCTCGCCCTTGCTCACCATttttgagcctgcttttttgtacaaacttgtgagcaaagtgtttcccaactgaaaaaaaaacaatttaatttaaaagtaagaagagtgcggacggtagagagaataaaagtcgaaaaaattatcctagaaacttctaatatttttaaaaattaaaaaaatataaaaagtgaggtcacaaataagacagttagaacatttgttgaaagcctaaaataaaaattcgactaaacagtgagaacaggaaaaacgctgggactgaatgtttgacctcattataaattcataaaatttaaatttcctaatctggataaaaacaaagataaaaacaaaataaaccaacaaaaaaccaacatgattagtcagatgaccagaaaactcacgttaatttcacaaggcccaaaaaaatctctccccctctcgttgctgcctgcacatctaactcctagcacgaaaatgtaccgtacaccatttcaaacactctcggcgggagtgttgcatactttttctctctggcagtttattttcaataaattcttcatttttatattgtataatgtctttattttatgaaaaattcattaatttaacagaaacaatggaagaaccaatggaagttgacaataaaagaccaaaggtgcTCGTGGGAATCACAACTGGTTCTGCAATTCCAATGACCAGAGTGAAATTGTAAAATTCGTA-3'
